# Microfluidic Flow-through SPME Chip for Online Separation and MS Detection of Multiple Analyses in Complex Matrix

**DOI:** 10.3390/mi11020120

**Published:** 2020-01-21

**Authors:** Yujun Chen, Tao Gong, Cilong Yu, Xiang Qian, Xiaohao Wang

**Affiliations:** 1 Tsinghua Shenzhen International Graduate School, Tsinghua University, Shenzhen 518055, China; clarajun@163.com (Y.C.); microfluid@sz.tsinghua.edu.cn (T.G.); wang.xiaohao@sz.tsinghua.edu.cn (X.W.); 2 College of Mechatronics and Control Engineering, Shenzhen University, Shenzhen 518060, China; yu@szu.edu.cn

**Keywords:** flow-through solid phase microextraction (SPME) on chip, quantitative analysis of flow-through solid phase microextraction (SPME), online separation, eluted, microfluidic chip, mass spectrometer (MS)

## Abstract

Simplifying tedious sample preparation procedures to improve analysis efficiency is a major challenge in contemporary analytical chemistry. Solid phase microextraction (SPME), a technology developed for rapid sample pretreatment, has flexibility in design, geometry, and calibration strategies, which makes it a useful tool in a variety of fields, especially environmental and life sciences. Therefore, it is important to study the coupling between the microfluidic electrospray ionization (ESI) chip integrated with the solid phase microextraction (SPME) module and the electrospray mass spectrometer (MS). In our previous work, we designed a solid phase microextraction (SPME) module on a microfluidic chip through geometric design. However, automation and calibration methods for the extraction process remain unresolved in the SPME on-chip domain, which will lead to faster and more accurate results. This paper discusses the necessity to design a micromixer structure that can produce different elution conditions on the microfluidic chip. By calculating the channel resistances, the microfluidic chip’s integrated module with the micromixer, SPME, and ESI emitters optimize the geometry structure. We propose the annular channel for SPME to perform the resistances balance of the entire chip. Finally, for SPME on a single chip, this work provides a quantitation calibration method to describe the distribution of the analytes between the sample and the extraction phase before reaching the adsorption equilibrium.

## 1. Introduction

The rapid and direct introduction of samples into a mass spectrometer (MS) to detect the target analytes in a complex sample matrix has led to the requirements of convenient, versatile sample preparation and pre-treatment methods. Different from the solid phase extraction (SPE) in which the analytes are absorbed completely from the sample matrix, followed by washing, elution, and concentration processes, SPME is an equilibrium-based extraction method that is proven to have high time efficiency and low sample consumption [1]. Diversiform SPME implementations were reported in the literature, mainly including an open-bed apparatus such as fibers, stirrers, vessel walls, and suspended particles; but in-tube formations were also suitable [2].

Automation is the key requirement for easily co-operating the SPME pre-treatment method with other detection instruments. All those SPME implementations adopted an extraction step in which the analytes were absorbed by the SPME substrates or coatings; followed by a desorption step using thermal, laser, elution liquid, etc. [3]. The desorbed analytes can be ionized and introduced to the MS directly or be introduced through a tandem setup such as a liquid chromatograph mass spectrometer (LC-MS) [4,5,6]. A mechanical SPME auto-sampler system can significantly improve the automation and efficiency for fiber SPME with LC-MS [7]. A typical design came from PAS Technology, who built a multi-fiber SPME auto-sampler, which can be interfaced directly with LC equipment via a mechanical arm [8].

An integrated microfluidic system is another promising way for automating the SPE or SPME processes [9]. Lin and co-works proposed a novel microfluidic system, including the functions of cell culturing, drug injection, cell lysis, isotopic labeling, sample pretreatment, and ESI-MS detection, in which an on-chip micro-SPE column was used for sample desalting and purifying [10]. Wu et al. developed a nanofiber membrane coupled with a microfluidic system for in vivo micro-extraction. Gasilova et al. proposed a microfluidic SPE system with gradient elution [11]. More recently, Campos et al. proposed a pillar based microfluidic SPME chip for cfDNA extraction [12].

Based on our previous works on cutting polydimethylsiloxane (PDMS) ESI emitters [13] and integrated SPE channel [14] fabrication by the multilayer soft lithography method for deep-shallow structures [15], herein, we propose a fully disposable and integrated SPME-ESI chip, which combines an online mixing channel, a disk SPME chamber, and an ESI emitter. The calibration curves of a single analyte in different sample matrices are firstly acquired by this chip system, and different elution conditions for different analytes are also verified. By switching the elution condition by the mixing channel, online separation of different analytes is possible.

## 2. Materials and Methods 

### 2.1. Materials and Instruments

#### 2.1.1. Instruments

All the experiments were implemented on a tailor made platform for fixing and coupling the microfluidic chip to the LCQ Fleet mass spectrometer (Thermo Fisher Scientific, Waltham, MA, USA). A precision air pump (peak pressure 345 mbar, accuracy 0.1%) and flow detector for sample storage and transport were purchased from Fluigent (Paris, France). The high voltage DC power supply was purchased from Dongwen High Voltage Power Supply (Tianjin, China). The microscope (Eclipse TE 2000-U) for observing the mixing on chips was purchased from Nikon (Tokyo, Japan).

#### 2.1.2. Reagents and Chemicals 

Reserpine and clenbuterol hydrochloride were purchased from ANPEL (Shanghai, China). Methanol and acetonitrile were purchased from Merck (Darmstadt, Germany). Formic acid and ammonia were purchased from Aladdin (Shanghai, China). All the above chemicals were analytical reagent grade. Mimic urine was purchased from XingHeng Co., Ltd. (Dongguan, China). The juice was chosen as most common beverage on the market produced by Master Kong. The C18 solid phase extraction column used in the experiment was purchased from ANPEL Laboratory Technologies (Shanghai) Inc. (CNW, Shanghai, China). The diameter of extraction particles was about 40 µm to 63 µm.

#### 2.1.3. Preparation of Real Samples

The stock solutions of reserpine and clenbuterol hydrochloride in concentrations of 410 μM/L and 160 μM/L were prepared by dissolving a suitable amount of solid samples in the solution base, which was made of a 4:1 ratio of water to acetonitrile. The stock solution with the base solution was diluted to obtain a set of reserpine solutions with concentrations of 0.41 μM/L, 1.23 μM/L, 2.05 μM/L, 2.87 μM/L, 3.69 μM/L, and 4.1 μM/L, respectively. Clenbuterol hydrochloride solutions were obtained in the same manner with concentrations of 0.8 μM/L, 1.6 μM/L, 3.2 μM/L, 4.8 μM/L, 6.4 μM/L, and 8.0 μM/L. The same concentrations of reserpine and clenbuterol were also prepared in the complex matrices such as mimic urine and juice.

To achieve online separation of multiple analytes, the stock solutions of reserpine and clenbuterol were mixed and diluted in the complex matrix to obtain a set of mixed solutions with concentrations (reserpine: clenbuterol) of 0.41 μM/L and 3.2 μM/L, 0.41 μM/L and 6.4 μM/L, 0.82 μM/L and 3.2 μM/L and 0.82 μM/L and 6.4 μM/L.

To calibrate the microfluidic mixing channel, fluorescent rhodamine aqueous stock solution was prepared in a concentration of 10 μM/L and diluted with acetonitrile solutions to obtain 10%–90% of the initial concentration for comparison. To achieve online separation of multiple analytes, dilute stock solutions of reserpine and clenbuterol with the base solution were made up to obtain a set of mixed solutions with concentrations of 0.41 μM/L and 6.4 μM/L and 0.82 μM/L and 3.2 μM/L.

### 2.2. Device Structure and Fabrication 

#### 2.2.1. Chip Design

**Figure 1:**
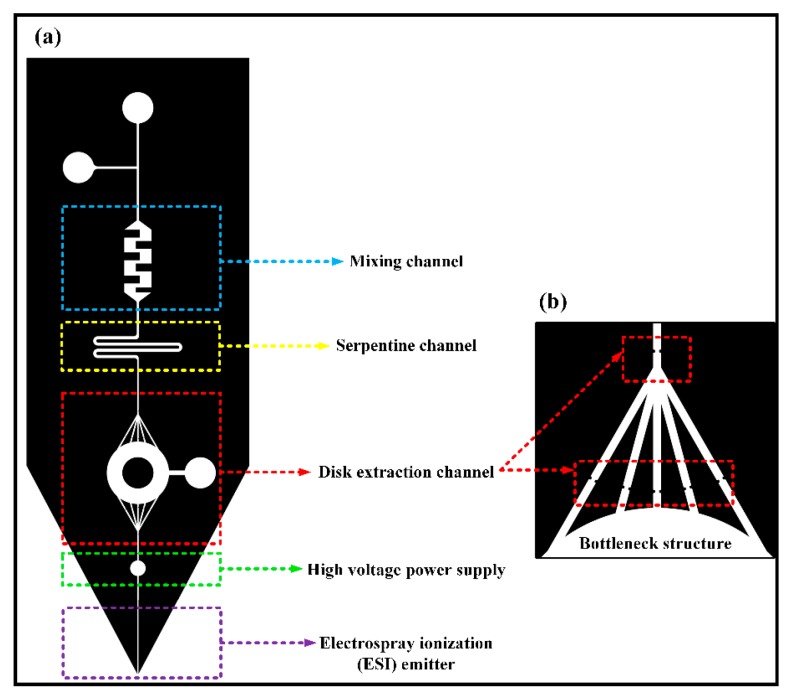
(**a**) Design of the chip structure; (**b**) bottleneck structure.

The optimization of the entire system changed the square extraction channel into a round one, which not only reduced the flow resistance of the extraction channels, but also improved the space utilization rate of filling more particles and absorbing more compounds. Meanwhile, the path would become longer than the origin. The microfluidic chip designed in this subject has a complicated structure. If a power supply is loaded on a steel needle at the upstream inlet of the chip, the voltage of the liquid flow is relatively unstable, which will cause the chip’s front-end electrospray to be unstable, and small droplets are likely to be generated. Moreover, the electrospray is not continuous, which affects the signal intensity of the coupled analysis of the chip and mass spectrometry, and directly affects the subsequent extraction quantitative results. Therefore, a circular area is designed between the solid-phase microextraction module downstream of the chip and the ESI nozzle to insert a steel needle. In the experiment, a crocodile clip is used to load the DC needle with high voltage to achieve the ESI spray function of the liquid sample. It avoids the influence of the complex structure of the chip front end on the electrospray. The microfluidic chip is illustrated in Figure 1. The chip structure consisted of five parts. The mixing channel was a rectangular channel with embedded obstacles, which was easy to handle and could mix liquids effectively. The serpentine channel could increase the length of the channel to help the liquids diffuse more evenly. The disk extraction channel was to fill the extraction particles, which could intercept particles with the bottleneck structure. A high voltage power supply was added between the disk extraction channel and ESI emitter.

#### 2.2.2. Chip Fabrication

**Figure 2:**
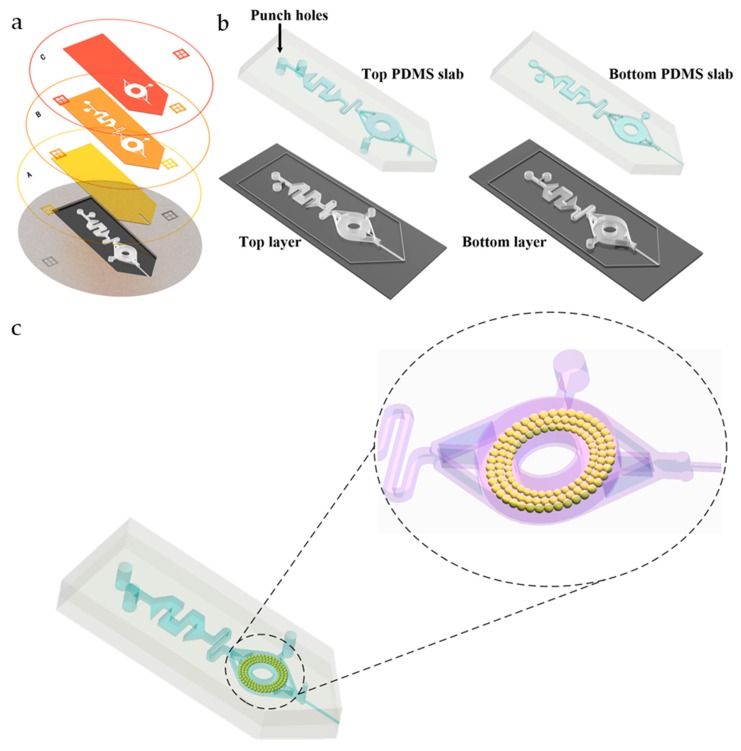
Manufacturing process of the microfluidic chip. (**a**) Photo Mask A shows the structure of the ESI emitter forming the features of 25 μm. Photo Mask B shows the structure of the mixing and extraction channel forming the features of 100 μm. Photo Mask C is to deepen the extraction channel forming the features of 100 μm. The bottom diagram shows the SU-8 master mold of the microfluidic chip. (**b**) Schematic diagram of the casting process of the PDMS top layer and bottom layer. (**c)** Schematic diagram of the bonding process of PDMS microfluidic chips and filling the C18 particles in the extraction channel.

The fabrication process was based on our previously developed multilayer soft lithography method for deep-shallow structures [13] and tip cutting [14], as shown in Figure 2. 

Briefly, three different layers were fabricated by lithography on a silicon wafer with an SU-8 negative photoresist by using Photo Masks A, B, and C, as shown in Figure 2a. Then, the top and bottom PDMS layers were die cast and applied with different layer depths, as shown in Figure 2b. In order to prevent the formation of droplets that may hinder the spraying, the depth of the bottom layer was about 25 µm; in order to improve the absorbing and storing ability, the depth of the top layer was about 225 µm. A 10:1 and a 5:1 mixed PDMS precursor and cross-linking agent were used for molding the top and bottom layer to achieve a strong and effective PDMS-PDMS bonding. After lithography and molding, a complete successive process including removing air bubble with a vacuum pump, curing, cutting, drilling, and bonding were performed. Sufficient detail is provided in the Supporting Information (Figure S1).

### 2.3. On-Chip Flow-through SPME Quantitative Method Work Principle

#### 2.3.1. Calculation of Distribution Coefficients

There are many traditional quantitative calibration methods for SPME, such as the external standard method, standard addition method, and internal standard method. Besides, there are many complex extraction calibration methods such as equilibrium extraction, exhaustive extraction, pre-equilibration extraction, and diffusion-based calibration.

The quantitative calibration method for the flow SPE mode we proposed, based on the equilibrium extraction system of SPME, required the analyte concentration to reach equilibrium between the sample matrix and solid phase extraction particles. In the traditional calibration methods for SPME, the distribution relationship of the target compound between the sample matrix and solid phase extraction particles can be described by Equation (1) according to the law of mass conservation.

(1) $$C_{0}V_{s}=C_{s}^{\infty }V_{s}+C_{f}^{\infty }V_{f}$$

where $c_{f}^{\infty }$ is the equilibrium concentration of the fiber coating, $c_{s}^{\infty }$ is the equilibrium concentration of the sample matrix, $V_{s}$ is the volume of the sample matrix, $V_{f}$ is the volume of the fiber coating, and $c_{0}$ is the initial concentration of the sample. 

The distribution coefficient of the target compound between the fiber coating and the sample matrix is defined as $k_{fs}$, expressed as follows:

(2) $$K_{fs}=\frac{C_{f}^{\infty }}{C_{s}^{\infty }}$$

The new quantitative calibration method for the flow SPE mode was based on an analysis system of a microfluidic chip coupled with mass spectrometry. According to the elution curve of MS, the concentration of the target compound solution could be quantified. As solid sorbent in the microfluidic chip adsorbing the target compound belonged to the total extraction method, in this case, in the flow SPE method, as the solution flowed through the extraction channel, the target compound was constantly adsorbed on the surface of the extraction particles, and the concentration of the target compound in the solution approached the initial concentration of the sample solution. The relationship between them is shown in Equation (3). When the adsorption was saturated, an expression of the equilibrium concentration and initial concentration was obtained by integrating Equation (3):

(3) $$C_{s}\left(t\right)=\left(1-e^{-\tau t}\right)C_{0}$$

(4) $$C_{s}^{\infty }=\frac{\int_{0}^{T}C_{s}\left(t\right)dt}{T}=C_{0}\left(1-\frac{1}{T\tau }\left(1-e^{-\tau T}\right)\right)$$

where $C_{0}$ is the initial concentration of the sample solution, $C_{s}^{\infty }$ is the equilibrium concentration of the target compound in the solution, τ is the time constant, and T is the extraction equilibrium time. According to the conservation of mass Equations (1) and (4), during a period of time, the following can be obtained:

(5) $$n_{f}\left(t\right)=\frac{C_{0}Q\left(1-e^{-\tau t}\right)}{\tau }$$

where Q is the velocity of the solution. When t≫T, to ensure that the adsorption saturation state is reached, $\left(1-e^{-\tau t}\right)$ approaches zero. Equation (5) can be simplified into the following equation:

(6) $$n_{f}^{\infty }=\frac{C_{0}Q}{\tau }$$

Then, the average adsorption concentration of the target compound on the surface of the extraction particles can be obtained:

(7) $$C_{f}^{\infty }=\frac{n_{f}^{\infty }}{V_{f}}$$

By combining Equations (2), (4), and (7), the distribution coefficient of the flow SPE mode under the state of extraction equilibrium can be obtained as follows:

(8) $$K_{fs}=\frac{C_{f}^{\infty }}{C_{s}^{\infty }}=\frac{n_{f}^{\infty }}{V_{f}C_{0}\left(1-\frac{1}{T\tau }\right)}$$

where $V_{f}$ is the total surface area of the extraction particles. 

#### 2.3.2. SPE Particle Filling and Activation

Reserpine can be adsorbed by C18 particles because of its alkaloid nature. Therefore, we selected C18 as the extraction particles. We soaked the extraction particles from the C18 SPE column in methanol to make a suspension. The suspension was injected from the third inlet (near the disk extraction channel) of the chip, and the extraction particles were automatically filled in the disc area and fixed by the bottleneck structure. First, we injected 200 μL of methanol solution in the extraction channel to activate the particles and then injected 200 μL of deionized water to rinse away the impurities. The average diameter of C18 particles was 60 μm. The number of C18 particles filling up the SPE channel was about 14,826, calculated as follows:

(9) $$S=14826*4\pi r^{2}=1.6768\times 10^{-4}m^{2}$$

#### 2.3.3. Chip Coupled MS Analysis System Construction

The experimental platform was mainly composed of five parts (Figure S2): MS, xyz-manipulator, precision air pump, flow detector (MFCS-EZ, FLUIGENT), and high voltage DC power supply. We placed the chip on the xyz-manipulator and adjusted the chip to the capillary inlet of the MS. The distance between the chip emitter and the MS inlet orifice was about 10 mm. The precision air pump and flow detector (MFCS-EZ) was mainly used to control the injection of the microfluidic chips. The voltage between the MS inlet orifice and the integrated ESI emitters was 5 kV.

## 3. Results and Discussion

### 3.1. Mixing on-Chip

**Figure 3:**
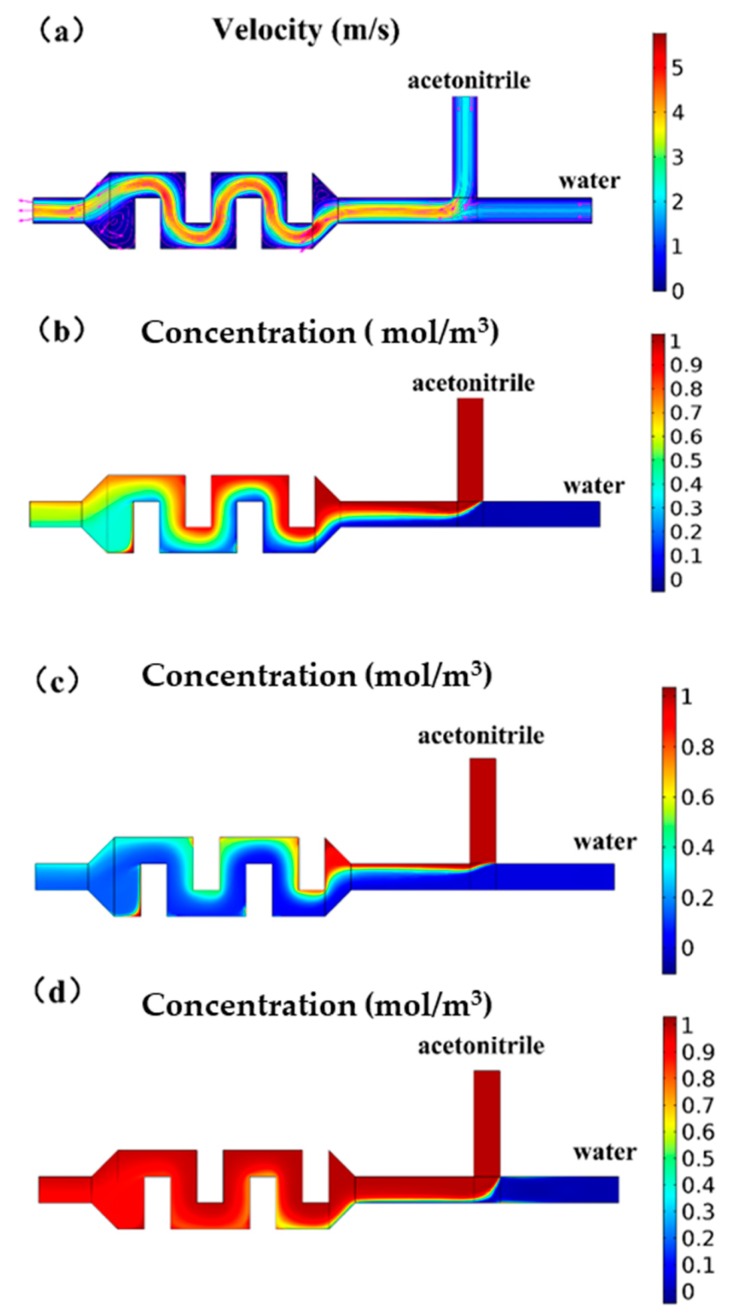
COMSOL simulation diagram of the chip mixing structure. (**a**) Schematic diagram of the velocity field when the inlet pressure ratio is 1:1. A vortex is formed at the inlet and outlet of the mixing channel to promote mixing. (**b**) Schematic diagram of the concentration field when the inlet pressure ratio is 1:1. (**c**) Schematic diagram of concentration field. By adjusting the pressure ratio, the concentration ratio of the mixed solution could reach 90%. (**d**) Schematic diagram of the concentration field. By adjusting the pressure ratio, the concentration ratio of the mixed solution could reach 90%.

The flow of the mixing channel was simulated by COMSOL, illustrating the flow field and concentration distribution (Figure 3). The mixing structure required effective mixing of acetonitrile and water at a concentration range of 10%–90%. It could be seen from the simulation results of the velocity field that when the pressure ratio was 1:1, the velocity field of the rectangular obstacle flow path generated vortices, as shown in Figure 3a, at the inlet and the outlet, which could help the liquid mix well. When the pressure was the same, observing the velocity field simulation at the T-type inlet, it could be found that the flow rate of acetonitrile was faster than water, because the liquid viscosity of acetonitrile was smaller and the same pressure value was applied, while the flow resistance of acetonitrile in the microchannel was smaller. It could be seen from the concentration field simulation results that although the pressure ratio was 1:1, because the two liquids had different viscosities, the flow resistance in the microchannel was different, so the liquid was not mixed 1:1, and the concentration of the mixed solution was close to 60%. The simulation results showed that the rectangular obstacle mixing flow channel could achieve the mixing function. The liquid at the outlet of the mixing flow channel was basically uniformly mixed, but there were some places with an uneven concentration distribution at the edge of the flow channel. We added a section of serpentine flow channel after the material mixing flow channel to increase the flow and promote the liquid to spread evenly. 

We used the level setting method, the phase field method, or the mobile grid to track the mobile interface in detail. The level set and phase field methods used a fixed grid and solved other equations to track the interface position. The moving grid method solved the Navier–Stokes equation on a moving grid with boundary conditions to represent the interface. In this case, the equations for mesh deformation must be solved. Because one surface in the geometry was used to represent the interface between two fluids in the moving mesh interface, the interface itself could not be broken down into multiple discrete surfaces. This meant that the “moving grid” interface could not be applied to problems such as droplet formation in inkjet equipment (in these applications, a level set or phase field interface would be appropriate). All three physical interfaces supported compressible (Mach number, Ma <0.3) and incompressible laminar flow, where one or both fluids could be non-Newtonian (see the detailed steps in the Supporting Information).

The fluorescence was easily observed through the microscope. Rhodamine B was mixed with acetonitrile driven by the pump inside the chip. The serpentine channel increasing the chip length was designed to ensure sufficient mixing after injecting. All results were easily observed from the fluorescence.

**Figure 4:**
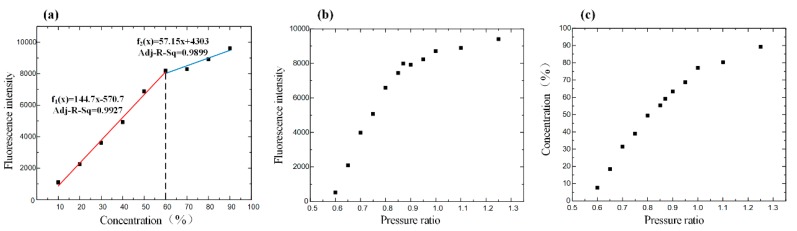
(**a**) Driving the standard fluorescence solution in the channel of the chip to obtain the relationship between fluorescence intensity and concentration. (**b**) Adjusting the pressure ratio to drive the solutions of rhodamine B and acetonitrile mixing in the channel and observe the fluorescence intensity of the mixture solution under the scanning electron microscope. The curve shows the relationship between fluorescence intensity and pressure ratio. (**c**) The relationship between the concentration and pressure ratio.

The average gray value as a symbol of the intensity was calculated by inputting the obtained picture, captured from 10%–90% rhodamine B standard mixing solution, into MATLAB. The linear relationship between fluorescence intensity and concentration was good before the concentration of the solution was 60%. After a 60% concentration, due to the influence of solvent polarity on the excitation of rhodamine fluorescence, when the acetonitrile solution decreased, the wavelength of the fluorescence spectrum shifted to red, and the fluorescence intensity increased and then decreased. The fluorescence intensity revealed the relation with the mixing concentration and pressure. Finally, we could find the intuitive relation between concentration and pressure (Figure 4).

The experiment’s result showed that the structure of the chip was efficient to mix the eluents, while the relation between pressure and solution concentration corresponded to the simulated one.

### 3.2. Extraction Disk Flow Field Simulation

Due to the two channels’ injection, this would cause some undesirable effects, such as backflow would occur in the front channels because the resistance of the extraction channel increasing. 

**Figure 5:**
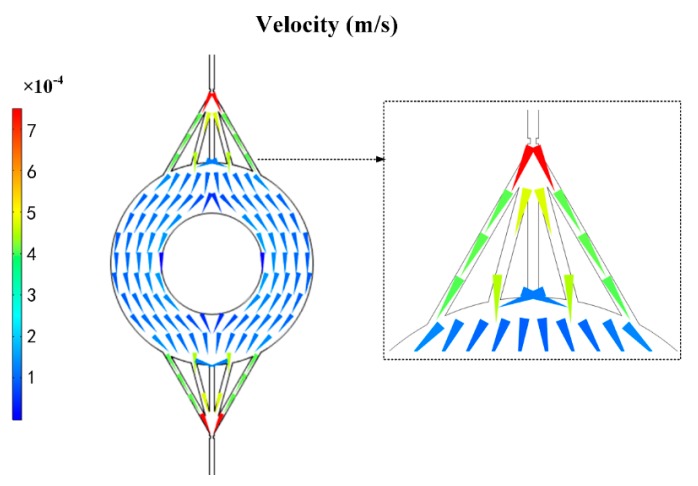
COMSOL velocity field simulation diagram of the chip disk shaped extraction channel.

Therefore, it was suggested that the flow field was evenly distributed in circular channels by simulating the circular channels’ flow field by COMSOL (Figure 5). This provided a method to absorb the particles sufficiently. Similar to Ohm’s law, the relationship of flow resistance, pressure drop, and flow rate is described as:

(10) $$R=\frac{P}{Q}$$

where P is the pressure drop, Q is the flow rate, and R is the flow resistance. The flow resistance was much lower in the circular channels compared to the rectangular channels (Table 1). As a result, the circular channels with multiple channels were the optimal choice because the decreased resistance and increased amount of C18 particles.

### 3.3. Determination of the Distribution Coefficient of the Single Compound under the Optimal Extraction Conditions in Different Sample Matrices (Water-Acetonitrile, Urine, Juice)

In the adsorption experiments, we used three kinds of reserpine solutions spiked in water-acetonitrile, urine, and juice, respectively, and clenbuterol hydrochloride as sample solutions. A precision air pump drove the sample solution from the outside into the channel of the chip. Then, when the solution passed through the SPE channel, the target compounds would be absorbed in the surface of the C18 and gradually reach the adsorption saturation status. To ensure the adsorption saturation status, 60 min was the optimal duration after comparing the adsorption curves. One-hundred-twenty millibars were applied to Inlets 1 and 2, with a flow rate of 5 µL/min.

In the desorption experiments, adjusting the injection pressure would cause a change in flow rate. Acetonitrile was injected into Inlet 1, and water was injected into Inlet 2; the pressure ratio between Inlet 1 and Inlet 2 was controlled at 0.95. When the solution passed through the mixing channels, it would obtain the optimal elution conditions, with 70% acetonitrile solution as the eluent. The pressure of the two inlets could be increased or decreased proportionally at any time to maintain the pressure ratio of 0.95, due to the unstable pressure of the air pump, which would not affect the solution’s concentration after mixing. Usually, when the flow rate was stable, the pressure at Inlet 1 was 100 mbar, and the pressure at Inlet 2 was 95 mbar. The adsorption and desorption process were recorded using MS.

Due to the optimal elution conditions for different samples, the eluent of reserpine was added with 1% formic acid in acetonitrile, while 5% aqueous ammonia was added to acetonitrile when eluting clenbuterol. The elution of the sample solutions of different concentrations was completed within almost 3 min. 

**Figure 6:**
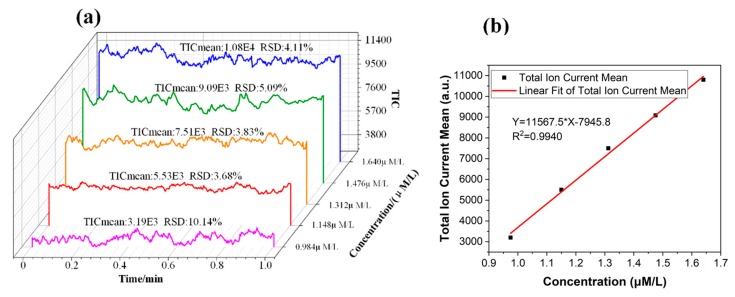
(**a**) The intensity signal of the reserpine solution with different concentrations. (**b**) The relationship between the concentration of the solution and the total ion current mean. TIC, total ion current.

To calibrate the extraction amount of the target compound by MS, the intensity of the elution signal needed to be converted into the solution concentration. A set of reserpine solutions with different concentrations was driven into the chip channel by the precision air pump. The ESI spray was formed on the tip of the chip for MS detection. The average intensity of the stable signal in one minute was used to calibrate the relationship between the concentration of the solution and the MS signal intensity, as shown in Figure 6. The total ion current (TIC) mean and relative standard deviation (RSD) were calculated and are marked in Figure 6a.

**Figure 7:**
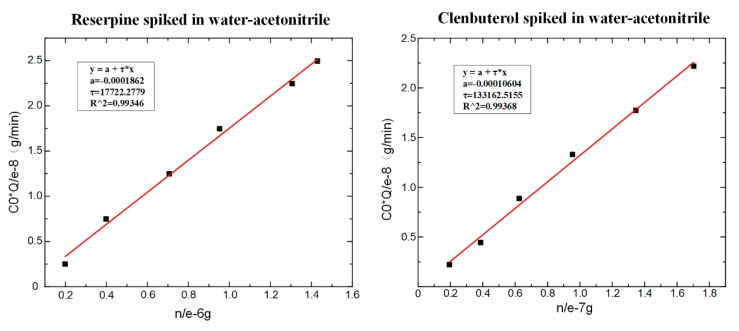
The fitting result of τ. (**a**) The time constant of reserpine. (**b**) The time constant of clenbuterol.

**Figure 8:**
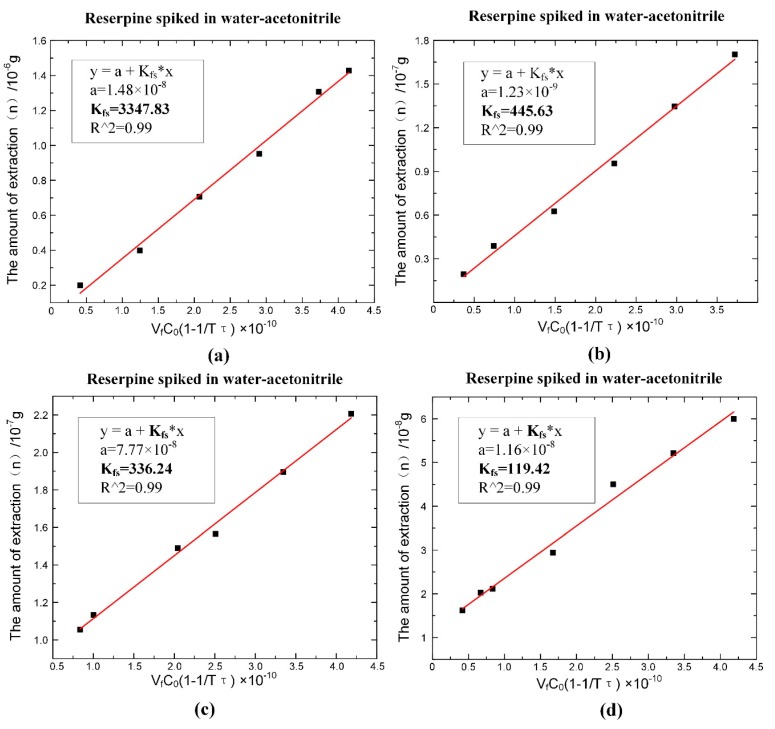
The fitting result of the distribution coefficient $k_{fs}$ of the single compound under the optimal extraction conditions. (**a**) $k_{fs}$ of reserpine spiked in water-acetonitrile. (**b**) $k_{fs}$ of clenbuterol spiked in water-acetonitrile. (**c**) $k_{fs}$ of reserpine spiked in urine. (**d**) $k_{fs}$ of reserpine spiked in juice.

According to Equation (6), the values of τ could be obtained by fitting, as shown in Figure 7. The fitting slope was the time constant τ, and it could be seen that the time constant of reserpine was 177.223 ($\tau_{R}=177.223{\;\min}^{-1}$) and the time constant of clenbuterol was 13,316.25 ($\tau_{C}=13316.25{\;\min}^{-1}$). Then, according to Equation (8), the distribution coefficient $K_{fs}$ could be obtained in the same way. The fitting curve of reserpine solutions spiked in water-acetonitrile is shown in the Figure 8a. In the curve, the slope was $k_{fs}$, and the fitted line had good linearity. The results indicated that the combination of reserpine and C18 particles in the flow SPE mode was consistent with the quantitative calibration method within the appropriate concentration range. Figure 8c, d shows the fitting curves of $k_{fs}$ in the urine samples and juice samples. The results revealed that $k_{fs}$ would decrease in the high salt complex sample matrix. $k_{fs}$ was fixed if the compound was in the same sample matrix, while in different sample matrixes, $k_{fs}$ needed to be remeasured for quantitative analysis. 

### 3.4. Separate Elution Experiment of Multiple Compounds under the Optimal Elution Conditions

The process of the adsorption experiment was the same as the distribution coefficient determination experiment. A mixed solution of reserpine and clenbuterol hydrochloride was pushed into the chip, and the target compounds were adsorbed using C18. When the mixed solution passed through the SPE channel, C18 would preferentially adsorb a large amount of reserpine, almost reaching a saturated state, and then adsorb the clenbuterol, because the binding capacity of reserpine and C18 was much better than clenbuterol [14]. In the desorption process, C18 would preferentially elute the clenbuterol and then elute the reserpine due to its weaker binding capacity. 

**Figure 9:**
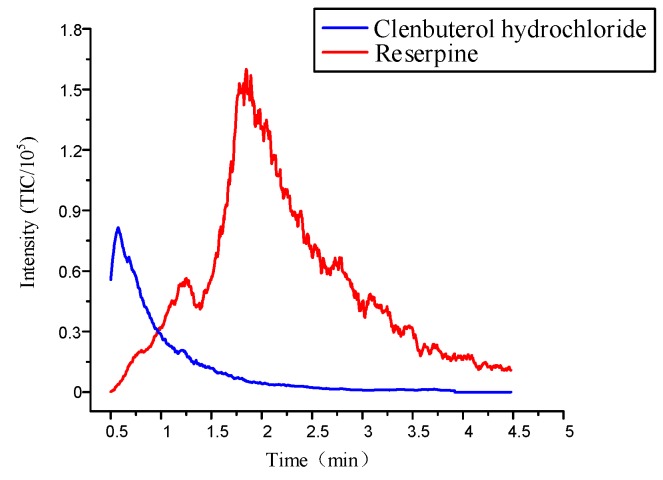
The elution curve of multiple compounds under the optimal elution conditions.

Previous studies showed that a 40% acetonitrile solution worked best in the individual elution of multiple compounds, because the polarity of elution solution could destroy the weaker combination and maintain a strong strength [14]. However, the microfluidic chips used in the previous study only had one inlet, which could only achieve a single concentration of elution condition. Optimizing the microfluidic chip by designing two inlets could achieve elution at different concentrations. This was similar for injecting acetonitrile into Inlet 1 and water into Inlet 2. Firstly, the pressure ratio between Inlet 1 and Inlet 2 was controlled at 0.75 so that the concentration of the acetonitrile solution reached 40% to desorb a large amount of clenbuterol hydrochloride. Figure 9 shows that when the mixed sample concentrations were 0.82 μM/L and 3.2μM/L, the intensity of clenbuterol gradually reached the maximum value, which was about $8.5\times 10^{4}$. Then, the intensity of clenbuterol decreased, and the intensity of reserpine gradually increased. When their intensities were almost the same, we adjusted the pressure ratio at 0.95 to desorb the reserpine. The intensity of reserpine gradually reached the maximum value, which was about $1.6\times 10^{5}$. Subsequently, the reserpine’s intensity decreased until the elution was complete. The result showed that the flow SPE microfluidic chip could elute two compounds separately.

However, simultaneously calibrating such a mixed solution based on our flow-through SPME chip was not as intuitive as the linear relationship between the amount of extracted analyte $n_{f}$ and the analyte concentration in the solution $C_{0}$. Unlike the SPE process in which there were enough absorption sites for almost all the analytes in the solution, SPME is an equilibrium extraction process by which only small analytes can be absorbed by limited sites; competition should be considered in such a situation. Assuming mixed analytes A (as reserpine) and B (as clenbuterol hydrochloride) could be eluted separately from our flow through SPME chip, just as the result shown above, the amount of extracted analyte $n_{fA}$ can be described as follows [16]:

(11) $$n_{fA}=\frac{K_{fsA}C_{0A}V_{s}\left(n_{f\max}-n_{fA}\right)}{\left(1+K_{fsB}C_{sB}^{\infty }\right)V_{s}+K_{fsA}\left(n_{f\max}-n_{fA}\right)}$$

All symbols in the above equation have the same meanings as shown in Equations (1)–(8) with an additional subscript “A” or “B” indicating the analyte; only one exception is that $n_{f\max}$ represents the total amount of adsorption sites. Obviously, it is a quadratic relationship between $n_{fA}$ and $C_{0A}$, and a unique solution with physical meaning can be acquired as [16]:

(12) $$n_{fA}=f\left(C_{0A},C_{sB}^{\infty }\right)\approx f\left(C_{0A},C_{0B}\right)$$

The approximate equal sign in the above equation was reasonable for the flow through system because under the equilibrium state, the final concentration of the analyte in the solution was almost the same as the initial one. A similar solution can be found for $n_{fB}$, and we can combine them in a vector form as:

(13) $$\left[\begin{array}{c} n_{fA}\\ n_{fB} \end{array}\right]=\vec{f}\left(\left[\begin{array}{c} C_{0A}\\ C_{0B} \end{array}\right]\right)$$

Assuming sufficient data $n_{fA}$,$n_{fB}$,$C_{0A}$,$C_{0B}$, such a system could be fit by neuronal network methods, which will be our future work.

## 4. Conclusions

The advantages of a microfluidic chip coupled mass spectrometry analysis system are high automation, high integration, real-time analysis, etc. Mass spectrometry is the best choice for quantitative analysis of unknown substances. It is a high sensitivity detection method. However, accurate quantitative analysis is still an important issue for microfluidic chips coupled to a mass spectrometry analysis system for solid phase microextraction.

In this study, we proposed a quantitative calibration method for the flow-through SPE mode spiked in the traditional SPME theory. This quantitative calibration method redefined the distribution coefficient $k_{fs}$ in the flow SPE mode and gave the determination method of $K_{fs}$ in the different sample matrices. Finally, by switching the concentration of the eluent on-chip, it was possible for two substances to be eluted under each optimal elution condition in the optimized microfluidic chip, and such operations could be made fully automatic by a peripheral control system. However, in our future work, a simultaneous calibration model of two or more analytes should be performed in this circulating SPME and optimized elution system. Such a flow-through SPME and optimized elution system shall be investigated out in our future works.

## Figures and Tables

**Table 1:** Determination of the flow resistance of the extraction channel. We filled the C18 particles into the extraction channel and then drove the water into the extraction channel. The flow rate was measured by the flow detector.

The Structure of Extraction Channel	The Amount of Filling C18 Particles	Pressure (mbar)	Velocity (µL/s)	Flow Resistance × 10^12^(Pa·s·m^−3^)
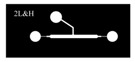	3130	50	0.1144	437.1032
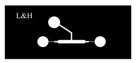	1565	50	0.3244	154.1221
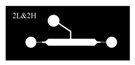	6260	50	0.1729	289.1183
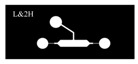	3130	50	0.2082	240.1085
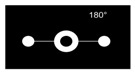	14,826	50	0.2046	244.3673
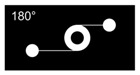	14,826	50	0.1797	278.2254
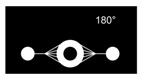	14,826	50	0.4162	120.1253

## Supplementary Materials

The following are available online at https://www.mdpi.com/2072-666X/11/2/120/s1: Figure S1: A schematic graph of the microfluidic chip made of PDMS, Figure S2: Microfluidic chip and mass spectrometry. (a) Microchip and mass spectrometry; (b) ESI spray of the microfluidic chip.
