# Supplementary material for: Microfluidic Flow-through SPME Chip for Online Separation and MS Detection of Multiple Analyses in Complex Matrix
Source: Micromachines (Basel). 2020 Jan 21;11(2):120. doi: 10.3390/mi11020120 (PMC7073986; doi:10.3390/mi11020120)
Supplement: Supplementary file 1 [file micromachines-11-00120-s001.pdf]

micromachines

MDPI

Supporting Information

Microfluidic Flow-through SPME Chip for Online Separation and MS Detection of Multiple Analyses in Complex Matrix

Yujun Chen [1,†], Tao Gong [1,†], Cilong Yu [2], Xiang Qian [1,*] and Xiaohao Wang [1]

[1] Tsinghua Shenzhen International Graduate School, Tsinghua University, Shenzhen 518055, China; clarajun@163.com (Y.C.); microfluid@sz.tsinghua.edu.cn (T.G.); wang.xiaohao@sz.tsinghua.edu.cn (X.W.)
[2] College of Mechatronics and Control Engineering, Shenzhen University, Shenzhen 518060, China; yu@szu.edu.cn
* Correspondence: qian.xiang@sz.tsinghua.edu.cn; Tel.: +86-755-2603-6755
† These authors contributed equally to this work.

Received: 6 December 2019; Accepted: 10 January 2020; Published: date

1. Experimental Section

1.1. Chip Fabrication Process

Polydimethylsiloxane (PDMS) is a widely used elastic material with simple processing technology and could be prepared repeatedly and in large quantities. The main steps of PDMS for preparing microfluidic chips are: liquid preparation, vacuum defoaming, pouring silicon wafers, vacuum defoaming, heating and baking, peeling and demolding, cutting and punching, oxygen plasma surface treatment, bonding, and baking setting. The specific process flow is as follows:

(1) Liquid formulation: PDMS is composed of a base liquid and a curing agent. By mixing base liquids with different proportions of the curing agent, PDMS with different elasticity and hardness can be obtained, and the crosslinking strength is higher when PDMS with different proportions are bonded. Therefore, in the experiment, the PDMS of 1:10 and 1:5 were used to prepare the upper and lower slices of the microfluidic chip. We used an electronic balance to weigh PDMS accurately with a ratio of 1:10 and 1:5 and used a glass rod to stir for 3 minutes until the base liquid and the curing agent were uniformly mixed.

(2) Vacuum defoaming: We put the agitated PDMS in a vacuum dryer and vacuum defoamed it for about 20 minutes until there were no obvious bubbles inside the PDMS. We took out the PDMS and blew off the surface bubbles with an ear wash ball.

(3) Casting silicon wafer: We placed the silicon wafer template in a Petri dish and poured 1:5 PDMS onto the silicon wafer template under the chip, with a thickness of about 7 mm; we poured 1:10 PDMS under the chip. On the silicon wafer template, the thickness was about 10 millimeters. If liquid leakage occurred at the chip inlet, the thickness of the PDMS on the wafer could be appropriately increased.

(4) Vacuum defoaming: We put the silicon wafer template with PDMS into a vacuum desiccator and vacuum defoamed it for about 20 minutes until there were no obvious air bubbles inside the Petri dish.

(5) Heating and baking: We placed the Petri dish in a constant temperature drying oven at 80 °C for 1 hour and adjusted the Petri dish to a horizontal state to prevent uneven chip thickness.

(6) Peeling and demolding: We took out the Petri dish, cut PDMS along the edge of the silicon wafer template with a scalpel, and slowly peeled the PDMS from the silicon wafer.

(7) Cutting and punching: We used a blade to cut PDMS along the chip edge to obtain the upper and lower pieces of the microfluidic chip. A hole punch with a diameter of 0.75 mm was used to punch holes on the upper slice at the two injection ports, the packed particle inlet, and the inlet for applying high voltage.

(8) Oxygen plasma surface treatment: We cleaned the surface of PDMS with tape to remove surface dust. We put the PDMS flow path up, put it into an oxygen plasma cleaner, and took it out after 1.5 minutes of surface treatment.

(9) Bonding: We aligned the upper and lower pieces under the microscope, first aligning the bottleneck structure on the extraction disk, and then slowly adjusting the two ends of the chip to align all the structures.

(10) Baking and sizing: We placed the aligned microfluidic chip in a 120 °C constant temperature drying oven and baked it for 24 hours. If the pressure applied to the chip during the experiment was large, the baking time could be appropriately extended and strengthen the bonding of the chip.

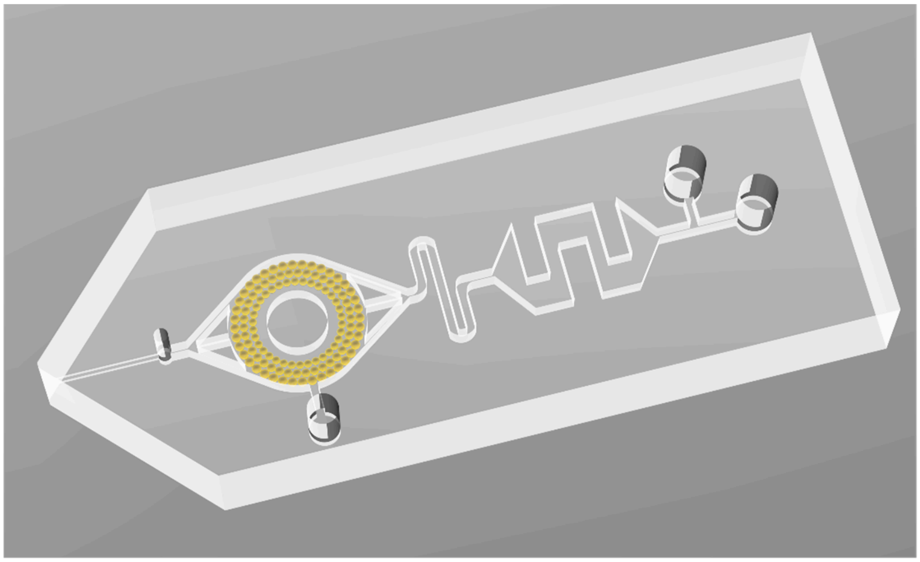

Figure S1. A schematic graph of microfluidic chip made of PDMS.

1.2. Sample Loading Process

We injected PDMS into a 200 μL pipette tip and baked in the oven until it solidified. It was used to block the particle inlet of the extraction channel. The experimental platform is shown in Figure S2. The microfluidic chip filled with particles was placed on a three-dimensional mobile platform, and the xyz axes were adjusted to align the ESI nozzle of the chip with the entrance of the mass spectrometer. The high voltage direct current was applied to the front end of the chip to form an electrospray. Both the No. 1 and No. 2 reservoirs were filled with standard sample solutions. The microfluidic drive control system was connected to the microfluidic chip. The No. 1 reservoir was connected to the No. 1 inlet, and the No. 2 reservoir was connected to No. 2 inlet. The loading solution was driven by the pressure pump from the storage tank into the liquid channel, and when passing through the solid phase extraction module channel, the target compound was adsorbed on the surface of the C18 particles, as the adsorption equilibrium was gradually reached. The loading times of C18 particles with different concentrations of the loading solution were different. The higher the sample concentration, the faster the particles reached the adsorption saturation state, and the shorter the extraction equilibrium time. In order to ensure that the loading process of the loading solution of each concentration could make the particle adsorption reach the adsorption saturation state, we compared the loading curve of each concentration and finally selected 60 min as the loading experiment time. The pressure applied to the chip liquid flow Channels 1 and 2 during the sample loading process was 100 mbar, and the flow rate was used for detection. At this time, the two phase flow rate was 5 μL/min. The voltage applied to the mass spectrometer inlet and the tip of the nozzle was 5 kV.

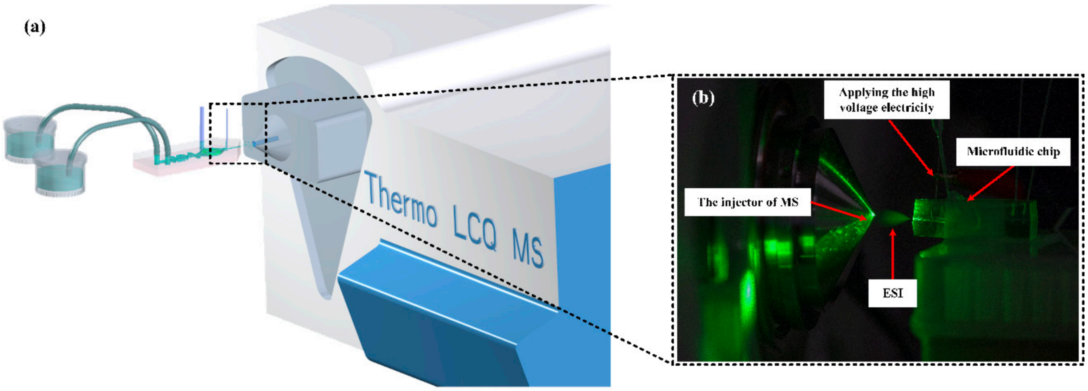

Figure S2. Microfluidic chip and mass spectrometry. (**a**) Microchip and mass spectrometry; (**b**) ESI spray of the microfluidic chip.

2. Simulation

2.1. Micro-Fluidic Chip Hybrid Structure Simulation

We used COMSOL to simulate the velocity field and concentration field of a rectangular obstacle flow path. The mixing structure required effective mixing of acetonitrile and water at a concentration range of 10%–90%. COMSOL Multiphysics is a general multi-physics coupling analysis simulation software, and users can easily set up multi-physics simulation analysis. In fluid analysis, a new microfluidics module was added to the software, which could control and analyze the fluid behavior at the microscale and could design micro-systems to simulate fluid flow. The microfluidics module contains many preset physics interfaces, such as single phase flow, thin-film flow, multiphase flow, porous media flow, and fluid-solid coupling. The module also provides level sets and phases for simulating two phase flow models, as well as field and moving grid research methods.

Because the rectangular obstacles had the same depth, the COMSOL simulation model was a two-dimensional model. To study the mixing performance of microfluids, the physical field selected was the mixture model in multiphase flow. Due to the low Reynolds number, the mixture of acetonitrile and water in the flow channel was in a laminar state. In the laminar state, the mass transfer was mainly affected by the diffusion when the two were mixed, and the physical field of chemical substance transfer needed to be coupled for simulation analysis. The physical field of chemical substance transfer selected was the transfer of rare substances for transient simulation research.

(1) Geometric construction: We set the length unit as mm and imported the mixed structure model.

(2) Material: We added water material properties from the material library; then added empty materials manually and acetonitrile properties to the material properties. More important in the mixed model were density and viscosity parameters. We set the density parameter of acetonitrile to 790 kg/m^3 and the dynamic viscosity parameter to 3–4 Pa s. We set up the domains where the two materials flowed.

(3) Mixture model (laminar flow) physics settings: The dispersed phase of the physics model selected was droplets/bubbles; the slip model was homogeneous flow; the properties of the continuous phase and the dispersed phase in the mixture property settings were from the domain material; the mixture selected was the Krieger type for the viscosity model; we added the inlet interface corresponding to water and acetonitrile and selected the pressure in the boundary conditions of the mixture. We set the pressure value of both inlets to 2 × 10^5 Pa. This limited the inlet pressure ratio to 1:1, and we selected the suppress the reflux option; we added the mixture outlet interface and also selected the pressure in the mixture boundary conditions and set the outlet pressure to 0 Pa.

(4) Physical field settings for dilute substance transfer: Convection was selected as the transfer mechanism; acetonitrile was selected as the diffusion material in the transfer properties, and the diffusion coefficient was the default value; the inlet interface corresponding to water and acetonitrile was added, and the concentration of the water inlet interface was set to 0 mol/m^3. The boundary conditions selected were the concentration constraints. The concentration of the acetonitrile inlet interface was set to 1 mol/m^3. The boundary conditions selected were concentration constraints; we added the mixture outlet interface.

(5) Grid setting: We selected the physics control grid in the grid setting; we set the element size to thin.

(6) Transient calculation settings: At the microscale, the flow of microfluidics could achieve a stable effect at the time level of ms. Therefore, the time unit was selected as ms, and the time range was set to (0, 0.1, 10).

We set the simulation parameters and started the simulation calculation. The calculation results are shown in Figure 3. It could be seen from the simulation results of the velocity field that when the pressure ratio was 1: 1, the velocity field of the rectangular obstacle flow path generated vortices at the inlet and the outlet, which could help the liquid mixing. When the pressure was the same, observing the velocity field simulation at the T-type inlet, it could be found that the flow rate of acetonitrile was faster than water, because the liquid viscosity of acetonitrile was smaller, and the same pressure value was applied, so the flow resistance of acetonitrile in the microchannel was smaller. It could be seen from the concentration field simulation results that although the pressure ratio was 1:1, because the two liquids had different viscosities, the flow resistance in the microchannel was different, so the liquid was not mixed 1:1, and the concentration of the mixed solution was close to 60%. The simulation results showed that the rectangular obstacle mixing flow channel could achieve the mixing function. The liquid at the outlet of the mixing flow channel was basically uniformly mixed, but there were some places with an uneven concentration distribution at the edge of the flow channel. We added a section of serpentine flow channel after the material mixing flow channel to increase the flow and promote the liquid to spread evenly.

2.2. Microfluidic Chip Extraction Structure Simulation

The optimized disk shaped extraction flow channel needed to be simulated to analyze whether the flow field was uniformly distributed in the flow channel to prevent particles from being in the disk extraction flow channel. Due to the uneven distribution of the liquid flow field and the excessively fast flow rate, the test compound could not be fully extracted, which affected the subsequent quantitative analysis. The velocity field of the disk shaped extraction channel was simulated and analyzed using COMSOL. The same simulation model selected was the two-dimensional model.

Because the liquid flowed through the disc shaped extraction flow channel, it was already mixed uniformly, so it was not necessary to consider the fluid mixing model. The physical field was selected as unidirectional flow (laminar flow) for the transient simulation research.

(1) Geometric construction: We set the length unit as mm and imported the disc shaped extraction flow channel structure model.

(2) Material: We added material properties of water from the material library and set the flow through all domains.

(3) Laminar flow physical field settings: The physical field model selected was incompressible flow and no turbulence model; fluid properties were derived from the materials; we added the inlet interface, selected pressure in boundary conditions, set the inlet pressure value to 2×10^5 Pa, and chose the suppress backflow option; we added the outlet interface, selected pressure in the boundary conditions, and set the outlet pressure value to 0 Pa;

(4) Grid setting: We selected the physics control grid in the grid setting; we set the element size to thin.

(5) Transient calculation setting: We selected ms as the time unit and set the time range to (0,0.1,1).

© 2020 by the authors. Submitted for possible open access publication under the terms and conditions of the Creative Commons Attribution (CC BY) license (http://creativecommons.org/licenses/by/4.0/).
